# Adsorption of amikacin during continuous venovenous haemofiltration in a swine model of acute renal failure

**DOI:** 10.1186/cc14197

**Published:** 2015-03-16

**Authors:** CD Gomersall, Q Tian, D Reynolds, M Ip, G Choi, G Joynt

**Affiliations:** 1 The Chinese University of Hong Kong, Shatin, Hong Kong

## Introduction

*In vitro *studies suggest that there is significant adsorption of amikacin, netilmicin, gentamicin and tobramycin to polyacrylonitrile haemofilters. This occurs rapidly and has the potential to substantially reduce the peak aminoglycoside concentration, which will reduce efficacy [1]. However, whether significant adsorption occurs *in vivo *is unknown. We therefore carried out a controlled *in vivo *study of the effect of amikacin adsorption by polyacrylonitrile filters during haemofiltration, using a porcine model of acute renal failure.

## Methods

A porcine model of acute renal failure was created by bilateral ligation of the renal arteries and veins. Eight pigs underwent haemofiltration using a 0.6 m^2^ polyacrylonitrile filter, blood flow 200 ml/ minute, ultrafiltration rate 1,000 ml/hour. All ultrafiltrate was returned to the pigs via a separate venous catheter so that any elimination of amikacin by haemofiltration could only be due to adsorption. Another eight pigs underwent sham haemofiltration in which blood was pumped around a haemofiltration circuit without a haemofilter and without ultrafiltration. Both groups of pigs were given intravenous amikacin, 15 mg/kg body weight over 30 minutes, and blood samples were taken from the arterial limb of the haemofilter circuit at 0, 5, 10, 15, 20, 25, 30, 40, 50, 60, 75, 90, 105, 120, 150, and 180 minutes after the start of the amikacin administration to assay amikacin concentrations.

## Results

Post-distribution peak concentration of amikacin was slightly, but significantly, lower in the CRRT group than that in sham group (55.0 ± 4.5 vs. 61.1 ± 5.9 mg/l, *P *< 0.05).

## Conclusion

This study shows that the effect of adsorption by polyacrylonitrile haemofilters on *in vivo *amikacin peak concentrations is small, and less than would be expected from *in vitro *data.

## Acknowledgement

This work was supported by a grant from the Hong Kong Research Grants Committee, CUHK 4644/08M.
